# A decade of cigarette taxation in Bangladesh: lessons learnt for tobacco control

**DOI:** 10.2471/BLT.18.216135

**Published:** 2019-01-21

**Authors:** Nigar Nargis, AKM Ghulam Hussain, Mark Goodchild, Anne CK Quah, Geoffrey T Fong

**Affiliations:** a American Cancer Society, Inc., 555 11th Street NW, Washington, DC, 20004, United States of America.; b Department of Economics, University of Dhaka, Dhaka, Bangladesh.; c Department of Prevention of Noncommunicable Diseases, World Health Organization, Geneva, Switzerland.; d Department of Psychology, University of Waterloo, Waterloo, Canada.

## Abstract

Bangladesh has achieved a high share of tax in the price of cigarettes (greater than the 75% benchmark), but has not achieved the expected health benefits from reduction in cigarette consumption. In this paper we explore why cigarette taxation has not succeeded in reducing cigarette smoking in Bangladesh. Using government records over 2006–2017, we link trends in tax-paid cigarette sales to cigarette excise tax structure and changes in cigarette taxes and prices. We analysed data on smoking prevalence from Bangladesh Global Adult Tobacco Surveys to study consumption of different tobacco products in 2009 and 2017. Drawing on annual reports from tobacco manufacturers and other literature, we examine demand- and supply-side factors in the cigarette market. In addition to a growing affordability of cigarettes, three factors appear to have undermined the effectiveness of tax and price increases in reducing cigarette consumption in Bangladesh. First, the multitiered excise tax structure widened the price differential between brands and incentivized downward substitution by smokers from higher-price to lower-price cigarettes. Second, income growth and shifting preferences of smokers for better quality products encouraged upward substitution from hand-rolled local cigarettes (*bidi*) to machine-made low-price cigarettes. Third, the tobacco industry’s market expansion and differential pricing strategy changed the relative price to keep low-price cigarettes inexpensive. A high tax share alone may prove inadequate as a barometer of effective tobacco taxation in lower-middle income countries, particularly where the tobacco tax structure is complex, tobacco products prices are relatively low, and the affordability of tobacco products is increasing.

## Résumé

Le Bangladesh applique un fort pourcentage de taxe sur les prix du tabac (au-delà du référent habituel de 75% du prix de détail) sans pour autant avoir atteint le bénéfice sanitaire attendu de réduction du tabagisme. Cet article se penche sur les raisons pour lesquelles la taxation du tabac n'est pas parvenue à réduire le tabagisme au Bangladesh. En utilisant les données gouvernementales couvrant la période comprise en 2006 et 2017, nous avons relié les tendances de vente des produits du tabac taxés avec la structure des droits d'accise sur le tabac et avec l'évolution des prix et des taxes sur le tabac. Nous avons analysé les données relatives à la prévalence du tabagisme à partir des enquêtes sur le tabagisme des adultes (GATS) réalisées en 2009 et 2017 au Bangladesh afin d'étudier la consommation des différents produits du tabac. À partir des rapports annuels des fabricants de tabac et d'autres ressources, nous avons examiné l'évolution du marché du tabac, côté demande et côté offre. Outre le fait que les cigarettes sont devenues plus abordables au fil du temps, trois facteurs semblent avoir sapé l'efficacité de l'augmentation des prix et des taxes dans l'objectif de réduction de la consommation de tabac au Bangladesh. Premièrement, la structure multi-niveau des droits d'accise sur le tabac a eu pour effet d'augmenter le différentiel de prix entre les marques, ce qui a poussé les consommateurs à opter pour des cigarettes moins chères. Deuxièmement, l'augmentation des revenus et le changement de préférence des consommateurs en faveur de produits de meilleure qualité ont fait que les consommateurs ont délaissé le tabac à rouler local (*« bidi »*) pour se tourner vers les cigarettes industrielles les moins chères. Troisièmement, du fait de l'expansion de ses marchés et de sa stratégie de différenciation des prix, l’industrie du tabac a fait évoluer les prix relatifs afin que les cigarettes les moins chères restent abordables. Un fort pourcentage de taxation peut s'avérer inapproprié, à lui seul, en tant que baromètre de l'efficacité des mesures de taxation du tabac dans les pays à revenu faible ou intermédiaire, notamment lorsque la structure de taxation du tabac est complexe, que les prix des produits du tabac sont relativement bas et qu'ils deviennent plus abordables au fil du temps.

## Resumen

Bangladesh ha alcanzado una elevada cuota de impuestos en el precio de los cigarrillos (superior al 75 % de referencia), pero no ha logrado los beneficios para la salud esperados de la reducción del consumo de cigarrillos. En este artículo exploramos por qué los impuestos sobre los cigarrillos no han logrado reducir el consumo de cigarrillos en Bangladesh. Utilizando los registros del gobierno entre 2006 y 2017, vinculamos las tendencias de las ventas de cigarrillos pagados con la estructura de los impuestos al consumo de cigarrillos y los cambios en los impuestos y precios de los cigarrillos. Se analizaron los datos sobre la prevalencia del tabaquismo de la Encuesta Mundial del Tabaco en Adultos de Bangladesh para estudiar el consumo de diferentes productos de tabaco en 2009 y 2017. Basándonos en los informes anuales de los fabricantes de tabaco y otras publicaciones, examinamos los factores de la demanda y la oferta en el mercado de cigarrillos. Además de la creciente asequibilidad de los cigarrillos, tres factores parecen haber socavado la eficacia de los aumentos de impuestos y precios en la reducción del consumo de cigarrillos en Bangladesh. En primer lugar, la estructura del impuesto especial de varios niveles amplió la diferencia de precios entre las marcas e incentivó la sustitución a la baja por parte de los fumadores, que pasaron de los cigarrillos de precio más alto a los de precio más bajo. En segundo lugar, el crecimiento de los ingresos y el cambio de las preferencias de los fumadores por productos de mejor calidad fomentaron la sustitución de los cigarrillos locales enrollados a mano (*bidi*) por cigarrillos de bajo precio hechos a máquina. Tercero, la expansión del mercado de la industria tabacalera y la estrategia de precios diferenciales cambiaron el precio relativo para mantener los cigarrillos de bajo precio baratos. Una elevada cuota de impuestos por sí sola puede resultar inadecuada como barómetro de los impuestos efectivos sobre el tabaco en los países de ingresos medios-bajos, en particular cuando la estructura de los impuestos sobre el tabaco es compleja, los precios de los productos del tabaco son relativamente bajos y la asequibilidad de los productos del tabaco está aumentando.

## ملخص

حققت بنغلاديش نسبة عالية من الضرائب في أسعار السجائر (بمعيار أكبر من 75٪) ولكنها لم تحقق الفوائد الصحية المتوقعة من انخفاض استهلاك السجائر. في هذه الورقة نستكشف لماذا لم ينجح فرض الضرائب على السجائر في الحد من تدخين السجائر في بنغلاديش. باستخدام السجلات الحكومية خلال الفترة من عام 2006 إلى عام 2017، فإننا نربط اتجاهات مبيعات السجائر مدفوعة الضرائب، بهيكل الضريبة المقتطعة على السجائر والتغيرات في ضرائب وأسعار السجائر. ﻗﻤﻨﺎ ﺑﺘﺤﻠﻴﻞ ﺍﻟﺒﻴﺎﻧﺎﺕ ﺣﻮﻝ ﻣﻌﺪﻝ ﺍﻧﺘﺸﺎﺭ التدخين ﻣﻦ ﺍﻻﺳﺘﻘﺼﺎﺀﺍﺕ العالمية المتعلقة ﺑﺎﻟﺘﺒﻎ بين ﺍﻟﻜﺒﺎﺭ في بنغلاديش ﻟﺪﺭﺍﺳﺔ ﺍﺳﺘﻬﻼك ﻣﻨﺘﺠﺎﺕ ﺍﻟﺘﺒﻎ المختلفة في الفترة من عام ٢٠٠٩ إلى ٢٠١٧. بالاعتماد على التقارير السنوية من شركات تصنيع التبغ والأدبيات الأخرى، نقوم بفحص عوامل جانب الطلب والعرض في سوق السجائر. بالإضافة إلى القدرة المزايدة على تحمل تكاليف السجائر، يبدو أن ثلاثة عوامل قد قللت من فعالية الضرائب وزيادة الأسعار في الحد من استهلاك السجائر في بنغلاديش. أولاً، قام هيكل الضرائب المقتطعة متعددة الأطراف بتوسيع فارق السعر بين العلامات التجارية، كما قام بتحفيز الاستبدال التنازلي بواسطة المدخنين من السجائر الأعلى سعراً إلى الأقل سعراً. ثانيا، شجع نمو الدخل وتغيير أذواق المدخنين تجاه منتجات ذات نوعية أفضل، على استبدال السجائر المحلية الملفوفة يدوياً (bidi) إلى السجائر ذات الأسعار المنخفضة المصنوعة آلياً. ثالثًا، أدى توسع السوق في صناعة التبغ واستراتيجية التسعير التفاضلية إلى تغيير السعر النسبي للحفاظ على أسعار السجائر منخفضة السعر. قد تكون الحصة الضريبية المرتفعة وحدها غير كافية كمقياس للضرائب الفعالة على التبغ في البلدان ذات الدخل المنخفض والمتوسط، خاصة عندما يكون هيكل ضريبة التبغ معقدًا، وأسعار منتجات التبغ منخفضة نسبيًا، والقدرة على تحمل تكلفة منتجات التبغ في ازدياد.

## 摘要

孟加拉国对卷烟价格（高于基准价格 75％）实施高税收份额，但此举并未降低卷烟消费量，实现预期的健康效益。本文中，我们探讨了孟加拉国烟草税未能成功减少吸烟的原因。根据 2006 年至 2017 年的政府记录，我们将已纳税卷烟的销售趋势与卷烟消费税结构以及卷烟税及其价格的变化联系起来。我们分析了孟加拉国全球成人烟草调查的吸烟率数据，旨在研究 2009 年和 2017 年不同烟草产品的消费情况。根据烟草制造商的年度报告和其他文献，我们研究了烟草市场的供求因素。除卷烟的可负担性增加之外，似乎还存在另外三大因素，削弱了税收和价格上涨在减少孟加拉国国卷烟消费方面的有效性。首先，多层消费税结构扩大了品牌之间的价格差异，并鼓励吸烟者从高价卷烟向下寻找廉价卷烟进行替代。其次，吸烟者的收入增长和对优质产品的偏好转变，鼓励其从手工卷制的本地卷烟 (*bidi*) 向上寻找机器制造的廉价香烟进行替代。再次，烟草行业的市场扩张和差别定价策略改变了相对价格，令廉价卷烟保持其价格优势。在中低收入国家，仅靠高税收份额不足以作为有效烟草税的晴雨表，特别是在烟草税结构复杂、烟草制品价格相对较低以及烟草制品的可负担性日益增加的情况下。

## Резюме

В Бангладеш достигнута высокая планка налогообложения сигарет (более 75% базисного показателя), но ожидаемой пользы для здравоохранения от сокращения потребления сигарет добиться не удалось. В данной статье рассмотрены причины, по которым налог на сигареты не привел к сокращению курения в Бангладеш. Используя правительственные данные за период с 2006 по 2017 год, авторы связали тенденции продаж облагаемых налогом сигарет со структурой акцизного налога на сигареты и с изменениями в налогообложении и ценообразовании на сигареты. Авторы проанализировали данные о частоте курения по данным Глобального опроса о потреблении табака взрослыми в Бангладеш, чтобы оценить потребление различной табачной продукции в период с 2009 по 2017 год. На основании ежегодных отчетов производителей табачных изделий и других справочных данных авторы изучили побочные факторы спроса и потребления сигарет на рынке. В дополнение к растущей доступности сигарет следующие три фактора снизили эффективность увеличения налогов и цен в вопросе снижения потребления сигарет в Бангладеш. Во-первых, многоуровневая структура акцизного налога увеличила ценовую разницу между брендами и стимулировала переход курильщиков с более дорогой продукции на более дешевую. Во-вторых, рост доходов населения и смещение предпочтений курильщиков в сторону более качественной продукции стимулировали переход с самодельных сигарет (так называемых биди) к дешевой фабричной продукции. В-третьих, расширение рынка табачной промышленности и дифференцированное ценообразование изменили относительный масштаб цен таким образом, что дешевые сигареты остались недорогими. Сама по себе высокая планка налогообложения может оказаться недостаточной для эффективного налогообложения табачных изделий в странах с малым и средним уровнем дохода. В частности, если налог на табачные изделия имеет сложную структуру, цены на табачные изделия остаются относительно низкими, а доступность табачной продукции растет.

## Introduction

Tobacco use is a major driver for the growth of noncommunicable diseases throughout the world.^1^^,^^2^ Raising the price of tobacco products through taxation is a proven measure for reducing tobacco consumption and thereby improving public health.^3^^,^^4^ The World Health Organization (WHO) has recognized this measure as a best-buy intervention for the prevention and control of noncommunicable diseases.^5^ Bangladesh has been identified as a high-achieving country for several tobacco control measures, including tobacco taxation, health warning labels and anti-tobacco mass-media campaigns.^6^ Raising tobacco tax to at least 75% of the retail price is considered a benchmark for best practice by WHO.^6^ As of 2016, only 32 countries globally, including Bangladesh, enforced such a high level of tax on tobacco products.^6^ The high level of achievement for Bangladesh in cigarette taxation is, however, contradicted by an increase in per capita cigarette consumption.

When cigarette prices increase, total cigarette consumption is expected to decrease. An increase in consumers’ income, on the other hand, can increase their ability to purchase more cigarettes and expand cigarette demand. The net effect on cigarette demand depends on the relative strength of the price and income increases which, in turn, affect the affordability of cigarettes. A recent study confirmed that cigarettes indeed became more affordable in Bangladesh between 2009 and 2015, and this change has been linked to the increase in cigarette consumption.^7^

In this paper, we examine the shifts in the tobacco product market in Bangladesh that might have contributed to the growth in cigarette consumption. We identify factors from both the demand and supply sides of the cigarette market that could have undermined the effectiveness of taxation in reducing cigarette smoking. Such factors can inform other countries’ tobacco taxation efforts.

## Cigarette tax and sales trends

The National Board of Revenue of the Bangladesh finance ministry applies a tiered excise tax (i.e. a supplementary duty) at four different rates for low-price, medium-price, high-price and premium brands of cigarettes. The excise tax rates are *ad valorem* (calculated as a percentage of the value of the product) and are successively higher for higher-priced brands belonging to the four price tiers. Table 1 shows the tax rates and range of post-tax retail prices recommended by the Board for different tiers from fiscal years 2006–2007 to 2017–2018. For example, a pack of 10 low-price cigarettes was taxed at 32% of the recommended retail price in the 2006–2007 fiscal year and medium-price cigarettes were taxed at 52% of the recommended retail price, and so on. The recommended retail prices and corresponding tax rate for each price tier are adjusted annually by the government. As shown in Table 1, the recommended retail prices and tax rates for each price tier gradually increased over time, with the highest increase in the low-price tier. Between 2006–2007 and 2014–2015, the increase in excise tax rate was 11, 8, 6 and 4 percentage points for low-price, medium-price, high-price and premium brands, respectively, over the same period.

**Table 1:** Excise tax rates and recommended retail prices by price tiers for cigarettes in Bangladesh from 2006–2007 to 2017–2018

Fiscal year	Low tier				Medium tier				High tier				Premium tier		
	Price per pack of 10, BDT	Excise tax, % of price	Total tax share, % of price		Price per pack of 10, BDT	Excise tax, % of price	Total tax share, % of price		Price per pack of 10, BDT	Excise tax, % of price	Total tax share, % of price		Price per pack of 10, BDT	Excise tax, % of price	Total tax share, % of price
2006–2007	5.25–6.24	32	47		10.50–12.49	52	67		18.00–24.99	55	70		≥ 30.00	57	72
2007–2008	6.00–6.99	32	47		12.50–13.49	52	67		19.00–26.49	55	70		≥ 35.00	57	72
2008–2009	6.50–7.50	32	47		13.25–14.25	52	67		21.00–28.00	55	70		≥ 41.00	57	72
2009–2010	7.25–8.75	32	47		16.25–17.25	52	67		23.25–29.25	55	70		≥ 46.25	57	72
2010–2011	8.40–9.15	33	48		18.40–19.00	53	68		27.00–32.00	56	71		≥ 52.00	58	73
2011–2012	11.00–11.30	36	51		22.50–23.00	55	70		32.36–36.00	58	73		≥ 60.00	60	75
2012–2013	12.10–12.30	39	54		24.75–25.25	56	71		35.20–39.50	59	74		≥ 66.00	61	76
2013–2014	13.69–13.91	39	54		28.00–30.00	56	71		42.00–45.00	59	74		≥ 80.00	61	76
2014–2015	15.00–16.50	43	58		32.00–35.00	60	75		50.00–54.00	61	76		≥ 90.00	61	76
2015–2016	18.00	49	64		NA	NA	NA		40.00–69.00	62	77		≥ 70.00	64	79
2016–2017	23.00	51	66		NA	NA	NA		45.00	63	78		≥ 70.00	65	80
2017–2018	27.00	53	68		NA	NA	NA		45.00	63	78		≥ 70.00	65	80

BDT: Bangladeshi taka; NA: not applicable.

Source: National Board of Revenue, Ministry of Finance, Government of Bangladesh.

Notes: In addition to excise tax (supplementary duty), the government applies value-added tax at 15% of the recommended retail price. The total tax share is given by the sum of excise and value-added tax rates. A 1% health development surcharge was included in the excise tax rates from 2014–2015. The official exchange rate (BDT per United States dollar) ranged from 68.93 in 2006 to 83.90 in 2018. In 2015–2016 the National Board of Revenue ruled that all brands belonging to the medium-price tier in 2014–2015 must move to the high-price tier, while no brands could move from the low-price to the medium-price tier. The following specific conditions were imposed: (i) all brands of cigarettes with current (2014–2015) retail price between BDT 32.50–35.00 to shift to BDT 40.00–69.00 tier; (ii) all brands of cigarettes with current retail price between BDT 50.00–54.00 to shift to BDT 70.00+ tier; (iii) all brands of cigarettes with current retail price BDT 90.00+ to add minimum BDT 10.00 to current retail price. The National Board of Revenue began to recommend single retail price in place of a price range in the low tier since 2015–2016 and in the high tier since 2016–2017.

In 2015–2016, the National Board of Revenue simplified the tax structure for cigarettes by eliminating the medium-price tier and repositioning medium-price products in the high-price tier with higher price and tax rate. Notably, the lower bound of the prices of the high-price and premium brands were lowered in 2015–2016 compared with 2014–2015 for repositioning the medium-price brands (Table 1). However, prices continued to increase for individual brands. 

Since 2014–2015, a 1% health development surcharge was included in the excise rates. In addition, there is a uniform 15% value-added tax based on the recommended retail price. In 2006–2007, the total tax share in the recommended retail price ranged from 47% for low-price brands to 72% for premium brands (Table 1). By 2017–2018, the tax share had increased for both tiers: 68% for low-price brands and 80% for premium brands. The total tax burden in this case includes only indirect taxes (excise and value-added tax) and excludes corporate income tax, which isolates the incidence of taxes on consumers from that on producers. The high achievement of Bangladesh in tobacco taxation was based on the total tax share of 78% of the recommended retail price of the most sold brand that belonged to the high-price tier in 2016–2017.

**Fig. 1:**
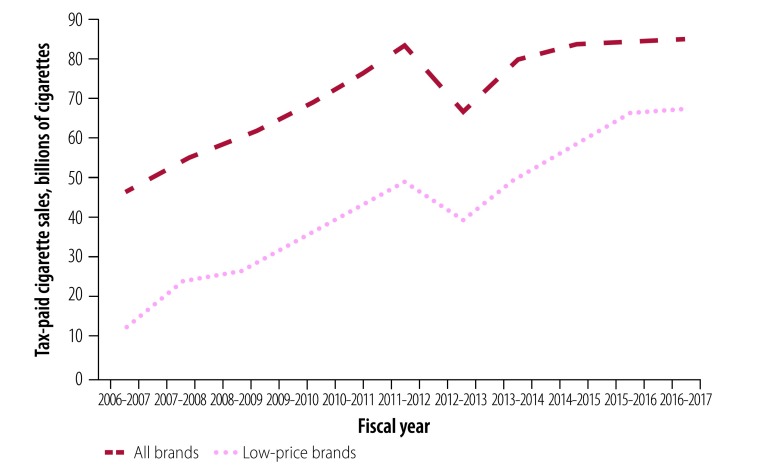
Tax-paid sales of all brands and low-price brands of cigarettes in Bangladesh from 2006–2007 to 2016–2017

Notes: Sales are the total number of tax-paid cigarettes sold in each fiscal year. We made calculations based on data from the National Board of Revenue, Ministry of Finance, Government of Bangladesh.

**Fig. 2:**
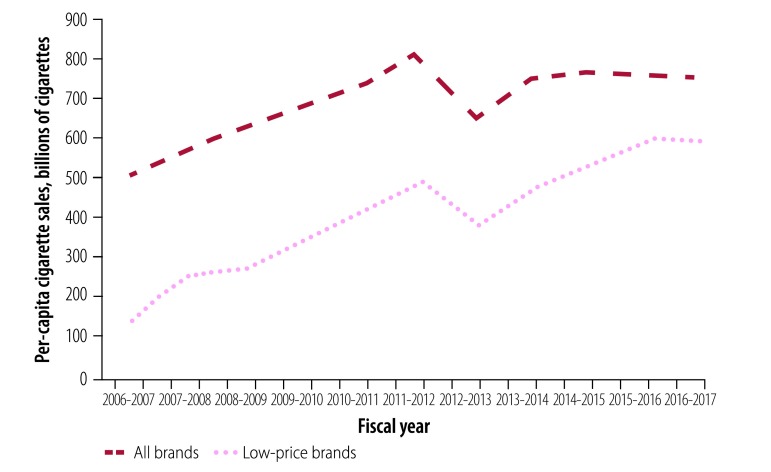
Per capita tax-paid sales of all brands and low-price brands of cigarettes in Bangladesh from 2006–2007 to 2016–2017

Notes: Per capita sales are the total number of tax-paid cigarettes sold in each fiscal year divided by the adult population (age 15+ years). We made calculations based on data on sales of cigarettes from the National Board of Revenue, Ministry of Finance, Government of Bangladesh and population data estimates from the United States Census Bureau International Data Base.^8^ Adult population of Bangladesh (age 15 years or older) in 2006: 91.5 million; 2007: 93.4 million; 2008: 95.2 million; 2009: 97.2 million; 2010: 99.3 million; 2011: 101.4 million; 2012: 103.6 million; 2013: 105.8 million; 2014: 108.0 million; 2015: 110.0 million; 2016: 112.0 million. The population estimate for 2016 differs from that which can be derived from the Global Adult Tobacco Survey prevalence data in Table 2 (19.2 million; 18.0% = 106.7 million).

Despite gradual increases in tax rates and prices across all brands, the volume of tax-paid cigarette sales in Bangladesh increased by 83% from 46.0 to 84.3 billion cigarettes between 2006–2007 and 2016–2017 (Fig. 1). This increase was driven by a 410% increase in low-price brands from 13.1 to 66.8 billion cigarettes. The growth was persistent in both absolute and per capita terms throughout the decade. Annual per capita cigarette sales for all brands increased by 49% from 503 to 752 cigarettes, while per capita sales of low-price brands increased by 317% from 143 to 597 cigarettes (Fig. 2). 

Note that the sales data presented here do not include illicitly traded cigarettes. Available evidence, however, suggests that illicit trade is very limited and is unlikely to make a significant impact on cigarette supply in Bangladesh. Between 2006 and 2016, illicit sales accounted for only 2.4 billion (4.0%) out of the total retail volume of 58.7 billion cigarettes sold in 2006 and 2.6 billion (2.9%) of the 88.7 billion cigarettes in 2016.^9^

## Understanding consumption trends

In view of the growing health burden that is expected to follow the upward trend in cigarette consumption, it is important to understand why cigarette taxation has not been an effective tool in reducing cigarette consumption in Bangladesh. Using government records over 2006–2017, we linked trends in tax-paid cigarette sales to cigarette excise tax structure and changes in cigarette taxes and prices that influenced brand substitution behaviour of smokers. We observed product substitution between 2009 and 2017 based on data on smoking prevalence from the Bangladesh Global Adult Tobacco Surveys. To study supply-side factors in the cigarette market, we examined annual reports and financial statements from the dominant tobacco manufacturers in Bangladesh and related literature on tobacco industry tactics (e.g. published research articles, online working papers and media reports). Specifically, we noted three critical patterns of change in consumers’ and producers’ behaviour: (i) brand substitution from higher-price to low-price cigarettes due to widening price differential between brands; (ii) product substitution from *bidi* (a cheap local hand-rolled smoked tobacco product) to low-price cigarettes due to income growth and shifting preferences of former *bidi* smokers; and (iii) tobacco industry pricing strategy that changed the relative price of cigarette brands in favour of low-price cigarette consumption.

### Brand substitution

**Fig. 3:**
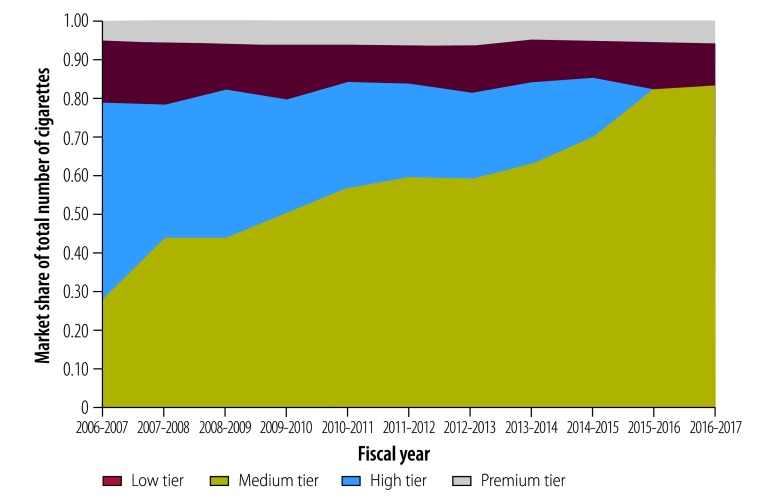
Market shares of low-, medium-, high- and premium-tier cigarette brands in Bangladesh from 2006–2007 to 2016–2017

Notes: Market shares are the annual number of tax-paid cigarette sold in each fiscal year in each tier divided by the total sales. We made calculations based on data from the National Board of Revenue, Ministry of Finance, Government of Bangladesh.

The disproportionately larger growth in low-price cigarette sales in Bangladesh led to a dramatic increase in the market share of these brands from 28% (13.1 out of 46.0 billion cigarettes sold) in 2006–2007 to 79% (66.8 out of 84.3 billion) in 2016–2017 (Fig. 3). This trend suggests substitution of low-price brands for higher-price brands at the population level, which can be attributed to the widening price gap between low-price and more expensive brands. Between 2006–2007 and 2014–2015, the price gap between the lower bounds of the recommended retail price ranges of medium-price and low-price brands increased (Table 1). The price gap became more pronounced after the repositioning of medium-price brands in the high tier in 2015–2016 and continued until 2017–2018, incentivizing the downward shift to cheaper brands while taxes and prices were increasing.

Tier-specific trends further revealed that while the market shares of high-price and premium brands remained steady throughout the decade, the market share of medium-price brands declined dramatically, until finally being eliminated in 2015–2016 (Fig. 3). These data suggest that the shift in the market composition occurred primarily from medium-price to low-price brands, even though the medium-price tier was merged with the high-price tier in the recommended price structure.

The multitiered complex excise tax structure in Bangladesh is chiefly responsible for the widening price differential among brands.^10^ Large price differentials discourage quitting by creating incentives for smokers to switch down to cheaper brands in response to tax and price increases.^11^^–^^13^ From the smokers’ point of view, there is not much product differentiation among brands other than price. Cigarettes can deliver nicotine in the same way irrespective of brand labels that are mostly engineered for marketing purposes. From the public health standpoint, all cigarettes are equally deadly.

### Product substitution

The growth in cigarette consumption with disproportionately larger growth in low-price cigarettes was seen in a period when Bangladesh recorded a large decrease in adult smoking prevalence from an estimated 23.0% (21.9 million smokers) in 2009 to 18.0% (19.2 million smokers) in 2017.^14^^,^^15^ This decrease is largely attributable to a reduction in the prevalence of *bidi* smoking from 11.2% to 5.0%, while cigarette smoking prevalence levelled off (14.2% in 2009 and 14.0% in 2017; Table 2). The volume of tax-paid *bidi* sales also reduced from 50.3 to 37.5 billion cigarettes, marking a 25% decline from 2006–2007 to 2016–2017, compared with an 83% increase in the volume of tax-paid cigarette sales (as mentioned above). The opposite trends in cigarette and *bidi* smoking prevalence and volume of sales indicate that there has been a structural shift in the composition of the smoked tobacco product market in Bangladesh.

**Table 2:** Adult smoking prevalence and breakdown of the adult smoking population in Bangladesh by type and use of smoked tobacco products in 2009 and 2017

Product type	No. of adult smokers, millions				Adult smoking prevalence, %		
	2009	2017	Change		2009	2017	Change
**Global Adult Tobacco Survey data**							
Cigarette (exclusive and dual)	13.5	15.0	1.5		14.2	14.0	−0.2
*Bidi* (exclusive and dual)	10.6	5.3	−5.3		11.2	5.0	−6.2
Others^a^	1.3	1.2	−0.2		1.4	1.1	−0.3
Overall	21.9	19.2	−2.7		23.0	18.0	−5.0
**Estimated data**^b^							
Cigarette only (exclusive)	10.0	12.7	2.7		10.5	11.9	1.5
*Bidi* only (exclusive)	7.1	3.0	−4.0		7.4	2.8	−4.6
Both cigarette and *bidi* (dual)	3.5	2.3	−1.3		3.7	2.1	−1.6

^a^ Other smoked tobacco products include hand-rolled cigarettes, pipes, cigars and waterpipes.

^b^ We made calculations based on adult smoking prevalence by product type and number of adult smoking population by product type obtained from the Global Adult Tobacco Surveys in Bangladesh in 2009 and 2017.^14^^,^^15^ In the absence of smoking prevalence data for other products in the Global Adult Tobacco Survey 2017, we have inputed it in the same proportion as in 2009.

Notes: *Bidi* is a cheap local hand-rolled smoked tobacco product. The adult populations of Bangladesh in 2009 and 2017 are not stated in the Global Adult Tobacco Survey reports, but can be estimated from the number of smokers and percentage prevalence of smoking.

The decline in *bidi* consumption can potentially contribute to the growth in cigarette consumption through migration of former *bidi* smokers to cigarette smoking and initiation of cigarette smoking in place of *bidi* smoking by the new smokers. A sizeable number of smokers use both cigarettes and *bidi,* and are prone to turn into exclusive cigarette smokers.^16^ Table 2 reveals that the number of exclusive cigarette smokers increased by 2.7 million between 2009 and 2017. However, sizable decreases in the number of exclusive *bidi* smokers and dual smokers led to a net decrease in the size of the smoking population by 2.7 million. Consequently, the share of exclusive cigarette smokers in the total smoking population increased from 45.7% (10.0 of 21.9 million smokers) to 66.1% (12.7 of 19.2 million smokers).

Between 2009–2010 and 2016–2017, the current price of a pack of 25 non-filtered *bidi* increased from 6.00 Bangladeshi taka (BDT) to BDT 10.61, while the current price of a pack of 10 cigarettes in the low-price tier increased from BDT 8.00 to BDT 23.00, widening the price gap between *bidi* and cigarettes. Contrary to the expectation that lower relative price of *bidis* would encourage *bidi* over cigarette smoking, the prevalence of *bidi* smoking decreased dramatically (Table 2). Associated research suggests that an increase in the affordability of cigarettes relative to *bidis* has driven this migration of *bidi* smokers to cigarettes.^7^ This rationale is more relevant for dual smokers who are likely to switch frequently between low-price cigarettes and *bidi* as close substitutes. The shift in the preference of smokers from hand-rolled *bidis* to higher-quality machine-made cigarettes can be linked to income growth. Demand for *bidi*, which may be perceived as inferior goods in the tobacco market, declined as the level of per capita income in Bangladesh increased at an annual average rate of 5% or above.^17^ Our observations are comparable to the shifting market shares in the Indonesian cigarette market from hand-made *kreteks* (the dominant tobacco product in Indonesia, which also includes cloves as a key ingredient) to machine-made *kreteks*.^18^

### Tobacco industry pricing strategy

The lower rate of excise tax for the low-price tier incentivized the expansion of the market for low-price cigarettes, which was historically dominated by two large domestic manufacturers: United Dhaka Tobacco Co. Ltd and Abul Khair Tobacco. The multinational giant British American Tobacco previously operated only in the market for higher-priced brands. In the late 2000s, it entered the market for low-price brands with the objective of addressing a broader array of consumer preferences and tapping the potential of market expansion.^19^ Despite the government’s effort to limit price competition by setting the recommended retail prices, British American Tobacco continued to capture the market in the low-price tier through the introduction of appealing new brands (e.g. Pilot introduced in 2009, Bristol in 2010 and Hollywood in 2011). Currently, the company operates as a dominant player with brands in all the price tiers and almost 60% share of the total cigarette market as of 2016 (50.0 out of the 84.3 billion cigarettes sold).

Under the price regulations discussed above, the ability of a dominant producer to extract profit only from the intensive margin (profit per unit) by increasing prices is limited. In these circumstances, producers can be induced to increase profit from the extensive margin as well through the expansion of market size. British American Tobacco recognized the role of volume growth in spurring higher profitability, alongside the contributions of better brand mix, price increases by government order, cost savings and productivity growth.^19^ The annual profit of British American Tobacco from cigarette sales in Bangladesh increased by 121% between 2009 and 2016, from 3.84 to 8.47 billion BDT in 2018 prices. The change is largely attributable to the volume growth of sales by 103%, from 24.7 to 50.0 billion cigarettes, driven by the increasing share of low-price brands to 74% (37 of 50 billion cigarettes) of the total production of the company by 2015.^19^

The government’s control of the recommended retail prices did not prevent manufacturers on the supply side from manipulating market prices to make financial gains. The comparison of market retail prices with the recommended retail prices by individual brands shows systematic deviation between the two for each price tier. This phenomenon takes place through retail channels and provides evidence in support of the market shift towards low-price brands. Associated research indicates that for low-price brands, market prices are lower than the recommended prices on average, suggesting the presence of discounting in the low-price tier.^20^ In contrast, the market prices are higher than the recommended prices for all the three higher price tiers.

The tobacco industry’s differential pricing strategy has thus changed the relative price in favour of low-price brands over higher-price brands. The strategy enabled the tobacco industry to maximize profit both from increasing prices at the high end of the market and expanding volumes at the low end. The strategy aligns with the global evidence that the cigarette industry tends to absorb tax increases on cheaper brands, while raising the price above the tax increase on more expensive brands.^21^^–^^30^

The multitiered cigarette taxation structure in Bangladesh, which was originally designed to favour domestic cigarette manufacturers producing low-price cigarettes, has ironically come to benefit the multinational giant British American Tobacco, who moved away from a business model dominated by premium products. Sustained growth in the annual profit and income largely from its expansion in the low-price cigarette market has appeared to inspire another multinational giant in the tobacco industry, Japan Tobacco International. This company has entered the cheaper cigarette market by acquiring the domestic manufacturer United Dhaka Tobacco, which is the second largest tobacco company in Bangladesh after British American Tobacco.^31^ The entry of Japan Tobacco is expected to intensify competition in the lower-price cigarette market that would create opportunities for a further increase in cigarette consumption. This business model of market expansion at the low end of prices conflicts with the public health interest of a country. 

## Lessons learnt

Sustained growth in cheap cigarette consumption can be devastating for public health for two reasons. First, the availability of cheap cigarettes encourages uptake among lower socioeconomic groups and youth, resulting in greater adverse health consequences on future generations, especially poorer people. Second, it enables smokers to switch down to cheaper brands rather than to reduce or quit smoking when taxes and prices are increasing. Together, these factors undermine the intended effect of tobacco tax increases.^32^^–^^35^

The current cigarette excise system has been in force for decades in Bangladesh. The tiered system poses an administrative burden on the tax authority for surveillance of the declaration of brands and the tax bases. Furthermore, market responses from both the demand and supply sides suggest that consumers are receiving the wrong incentive from relatively low prices and instead of quitting are increasing their consumption of cheap cigarettes.

Replacing the tiered excise tax structure in Bangladesh with a uniform system is therefore needed. This system would ideally include a major specific component (e.g. a lump sum tax per pack of cigarettes), creating a mixed or hybrid tax structure (both specific and *ad valorem*). While the specific component will help to reduce the price gap between low-price and higher-price brands and ensure a steady and predictable revenue flow to government, the *ad valorem* component will extract more revenue from the higher-price brands. A mixed tax structure has been implemented effectively by the European Commission in its Member States, with the application of a so-called tax floor (known as the minimum excise duty) to discourage consumption of low-price cigarettes.^36^ In the longer term, *ad valorem* taxes should be completely replaced by a uniform specific excise system. This system would avoid the additional burden of administering the recommended retail prices for cigarettes as the tax base for an *ad valorem* excise tax. In addition, the specific excise tax should be indexed to inflation and income growth to prevent the affordability of cigarettes from increasing. 

A well designed uniform excise tax system, with greater reliance on specific taxes, is key to effective tobacco control via taxation.^37^ Nevertheless, a large number of low- and middle-income countries levy differential tax rates based on prices and other product characteristics (e.g. packaging, volume of production, cigarette length or filter, country of origin).^4^ A complex tax structure with multiple tax rates creates an administration challenge for governments and jeopardizes revenue generation due to widespread tax avoidance among tobacco producers and consumers. This in turn limits the effect of tax increases on reducing tobacco consumption. These countries can potentially make major public health and revenue gains by simplifying their complex excise systems. For example, the landmark Sin Tax law, implemented in the Philippines from 2012 to 2017, simplified a four-tiered specific excise tax structure for cigarettes into a uniform specific system by increasing taxes and retail prices across all tiers, with larger increases at the lower tiers. These reforms contributed to a continuous decline in smoking prevalence.^6^ Reforming a complex tax structure, however, requires strong political will and people’s support. Evidence from the Philippines suggests that the allocation of tobacco tax revenue to improve equity in health and to provide financial protection to those affected by tobacco control measures can help overcome this political challenge.^38^

Besides the structural demand and supply-side forces, we have identified, there might be other factors that have contributed to the growing trend in cigarette consumption. Such factors could include tobacco industry tactics to undermine overall tobacco control initiatives (e.g. activities involving corporate social responsibility such as charity-giving to encourage smoking, particularly among youth) and weak enforcement of non-tax tobacco control measures (e.g. non-compliance of graphic health warnings on tobacco packaging) in the country.^39^^,^^40^ More research is needed into factors affecting trends in consumption of tobacco.

## Conclusion

High tax share alone may prove inadequate as a barometer of effective tobacco taxation in lower-income countries, particularly where the tobacco tax structure is complex, tobacco products prices are relatively low and the affordability of tobacco products is increasing. A simple and uniform specific tobacco tax structure, indexed to inflation and income growth, is a prerequisite for tobacco tax and price increases to be effective in reducing consumption of tobacco products.
